# Targeted Lysosomal Degradation of Extracellular and Membrane Proteins: From Receptor Hijacking to Programmable Endolysosomal Routing

**DOI:** 10.1002/advs.77675

**Published:** 2026-09-08

**Authors:** Ke Liu, Xiyan Wang, Xiaozhen Liu, Chaoqi He, Da Qian, Xuli Meng, Liquan Zhu

**Affiliations:** ^1^ School of Pharmacy Hangzhou Normal University Hangzhou Zhejiang China; ^2^ General Surgery Cancer Center, Department of Breast Surgery Zhejiang Provincial People's Hospital (Affiliated People's Hospital) Hangzhou Medical College Hangzhou Zhejiang China; ^3^ Key Laboratory for Diagnosis and Treatment of Upper Limb Edema and Stasis of Breast Cancer Hangzhou Zhejiang China; ^4^ Central Laboratory，Department of Burn and Plastic Surgery‐Hand Surgery Changshu Hospital Affiliated to Soochow University Changshu No.1 People's Hospital Changshu Jiangsu China

**Keywords:** axoplasmic transport, cell biology, endocytic cycle, extracellular, Internalization, membrane protein, protein degradation, transport protein, vesicle

## Abstract

Lysosome‐targeting chimeras (LYTACs) have emerged as a strategy for eliminating secreted, extracellular, and plasma‐membrane proteins by redirecting them to the endolysosomal system. By coupling target recognition to receptor‐mediated uptake, LYTACs exploit endogenous trafficking pathways to access disease‐associated proteins beyond the reach of conventional intracellular degradation mechanisms. Related target‐intrinsic and cross‐linking‐driven strategies can likewise promote lysosomal target delivery without recruiting a separate clearance receptor. However, target internalization alone does not establish productive degradation. Following entry, target‐containing complexes encounter a competitive endosomal network in which they may recycle, undergo retrograde transport or transcytosis, remain in non‐degradative compartments, or proceed to lysosomes. Here, we present a routing‐centered framework for understanding and designing extracellular and membrane‐protein degradation. We discuss how target biology, receptor choice, tissue distribution, ligand competition, signaling liability, molecular architecture, and intracellular sorting shape degrader performance. We also outline evidence standards for distinguishing bona fide lysosome‐dependent target loss from surface depletion, redistribution, epitope masking, shedding, secretion blockade, transcriptional effects, and nonspecific toxicity. Together, these principles define the transition from receptor hijacking to programmable endolysosomal routing.

## Graphical abstract

Targeted chimeric degraders engage extracellular or membrane proteins and endocytosable cell‐surface receptors, forming ternary complexes that induce receptor‐mediated internalization. Following endocytosis, post‐endocytic sorting—not internalization alone—determines target fate. Receptor selection, molecular valency and configuration, receptor clustering, cargo–receptor dissociation, and MVB/ILV entry promote either non‐productive recycling or retention in non‐degradative compartments, or productive delivery to lysosomes. Bona fide lysosomal degradation is validated by total protein loss, lysosome‐inhibitor sensitivity, receptor dependence, and intracellular trafficking analysis.

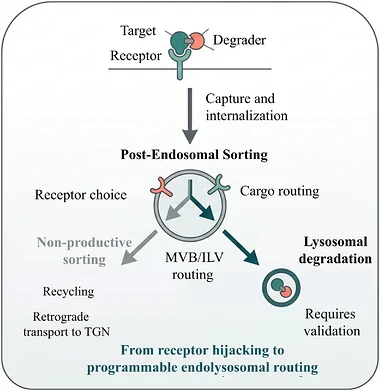

## 1 Introduction: From Receptor Hijacking to Programmable Endolysosomal Routing

**FIGURE 1:**
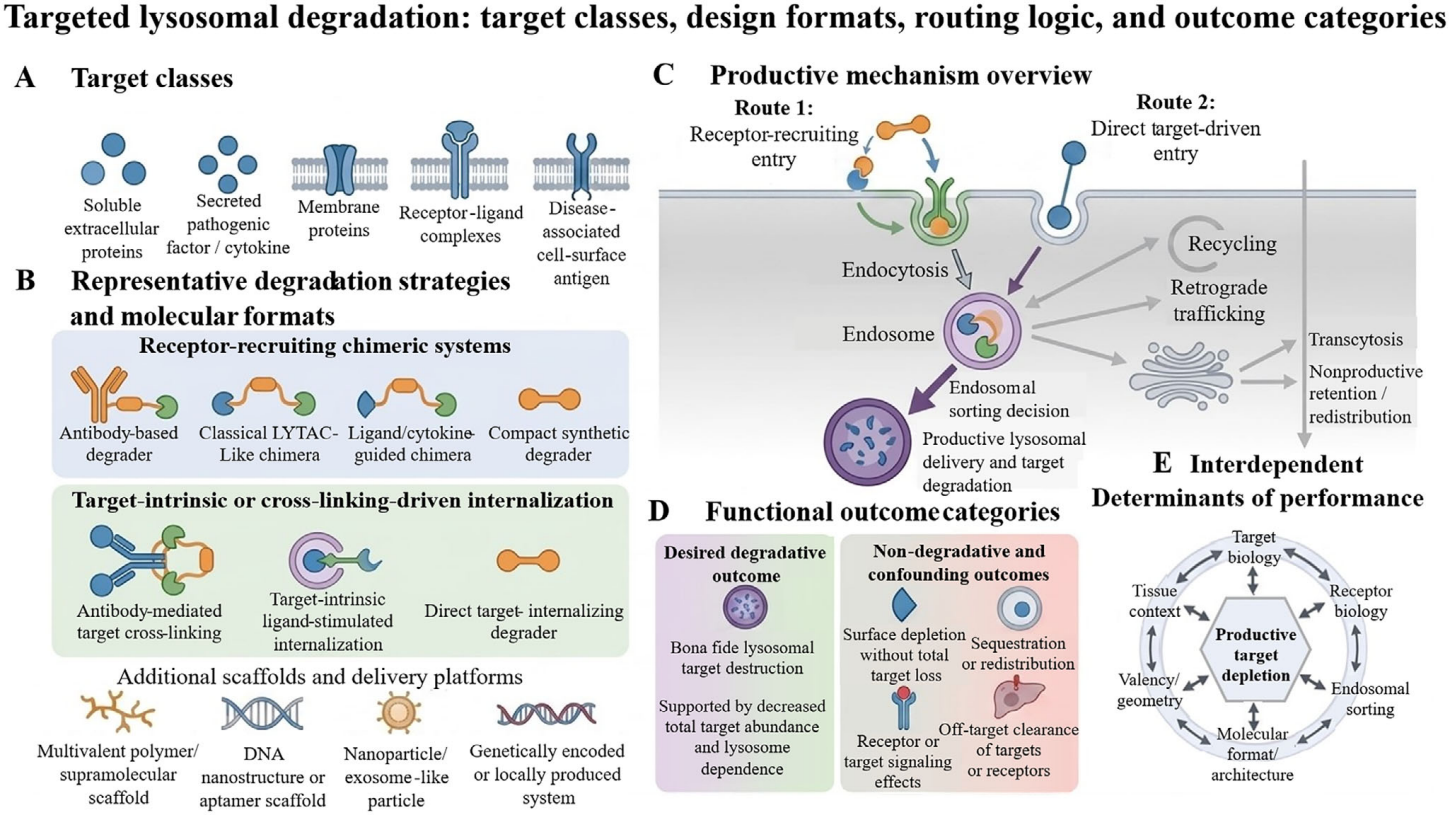
Conceptual framework for targeted lysosomal degradation across target classes, entry routes, molecular formats and functional outcomes. (A) Representative target classes include soluble extracellular proteins, secreted pathogenic factors or cytokines, membrane proteins, receptor–ligand complexes and disease‐associated cell‐surface antigens. These targets can enter degradative pathways through distinct mechanisms, including direct target‐driven internalization and receptor‐recruiting entry. (B) Representative targeted lysosomal degradation strategies and molecular formats include antibody‐based degraders, classical LYTAC‐like chimaeras, ligand‐ or cytokine‐guided chimaeras, compact synthetic degraders, multivalent polymers or supramolecular scaffolds, DNA nanostructures or aptamer‐based scaffolds, nanoparticles or exosome‐like particles, and genetically encoded systems. These formats differ in structural definition, valency, size, geometry, tissue access and intracellular trafficking behavior. (C) Productive lysosomal degradation requires more than target binding and cellular entry. Following direct target‐driven or receptor‐recruiting internalization, degrader–target complexes enter a competitive endosomal sorting network that may lead to recycling, retrograde trafficking, non‐degradative retention or progression toward lysosomes. (D) Functional outcomes should be distinguished mechanistically. Desired activity corresponds to bona fide lysosomal target destruction, supported by a reduction in total target abundance. Potentially confounding outcomes include surface depletion without total target destruction, intracellular sequestration or redistribution, receptor clearance, target‐signal perturbation, and other non‐degradative effects. (E) Degrader performance is determined by the interaction of target‐intrinsic biology, target cross‐linking, receptor biology, tissue and cellular context, molecular geometry, valency and endosomal sorting. Additional scaffolds and delivery platforms can further modulate these determinants. Thus, targeted lysosomal degradation is an integrated design problem in which molecular format, entry mechanism, trafficking fate and biological context jointly determine whether internalization results in productive target depletion.

Targeted protein degradation (TPD) has expanded therapeutic intervention from sustained target occupancy to selective elimination of disease‐associated proteins [1]. By reducing target abundance, degraders can suppress catalytic, scaffolding, and signaling functions that may persist despite conventional inhibition [1, 2, 3, 4]. PROTACs and molecular glues established this principle for intracellular proteins by recruiting E3 ubiquitin ligases to promote ubiquitin–proteasome‐dependent degradation [5, 6, 7, 8] (Figure 1A).

However, the proteasome acts principally in the cytosol and nucleus and is therefore poorly suited to many therapeutically relevant extracellular and membrane‐associated proteins [9, 10]. These targets include secreted factors, extracellular‐matrix‐associated proteins, receptor tyrosine kinases, immune checkpoints, cytokines, growth factors, adhesion receptors, transporters and viral entry factors [11] (Figure 1B). For these proteins, neutralization or inhibition may be incomplete because pathogenic factors can persist in extracellular compartments and membrane proteins can retain scaffolding, ligand‐independent signaling or recycling‐dependent re‐expression [12, 13, 14]. Their active elimination therefore provides a distinct pharmacological opportunity (Figure 1C).

Lysosomes are the principal degradative endpoint for extracellular cargo and cell‐surface proteins. LYTACs exploit endogenous receptor‐mediated endocytosis to redirect bound targets into the endolysosomal system. Early designs conjugated target‐binding antibodies to mannose‐6‐phosphate ligands that recruit the cation‐independent mannose‐6‐phosphate receptor (CI‐M6PR) [15]. Subsequent systems exploited the hepatocyte‐enriched asialoglycoprotein receptor (ASGPR), often using triantennary N‐acetylgalactosamine ligands, to enable liver‐biased uptake and degradation [16]. These studies established that induced proximity can couple extracellular and membrane targets to endocytic receptors, thereby extending TPD beyond intracellular E3 ligases [17, 18, 19].

Since then, lysosomal TPD has diversified across receptor systems and molecular formats, including antibody‐based chimeras, bispecific biologics, small‐molecule or ligand‐based constructs, aptamers, nucleic‐acid scaffolds and nanoscale assemblies [20, 21, 22]. The central challenge is not target internalization alone, but productive delivery to lysosomes. Following receptor engagement and uptake, target–degrader complexes may recycle [23], undergo retrograde trafficking or persist in non‐degradative endosomal compartments rather than reach lysosomes [24, 25, 26]. Accordingly, target loss should be established by measurement of total target depletion and lysosomal dependence; receptor dependence and trafficking analyses provide important mechanistic support for defining and optimizing the route of degradation in the relevant biological context [23].

**FIGURE 2:**
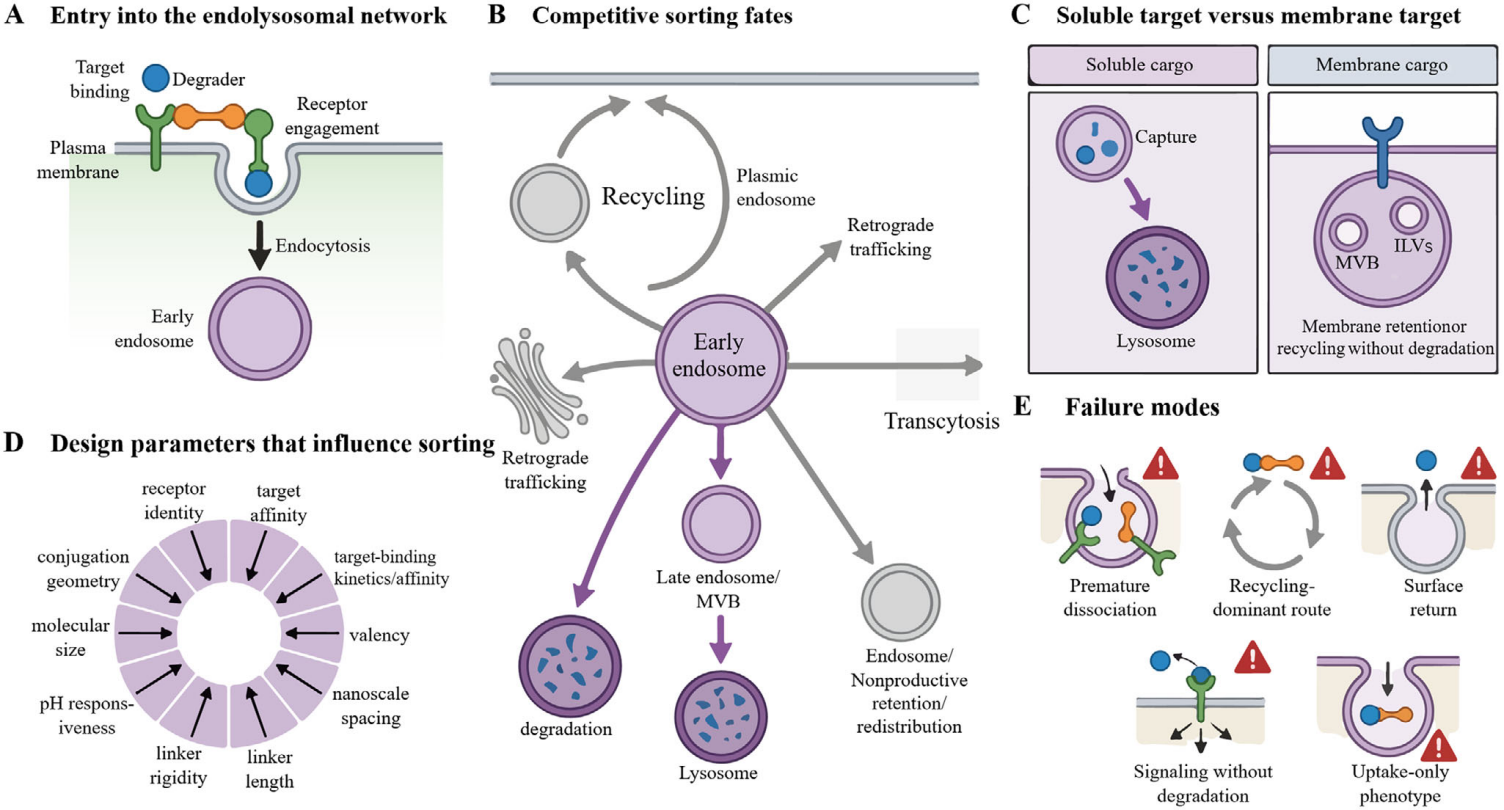
Post‐endocytic sorting determines whether proximity becomes productive degradation. (A) After simultaneous engagement of the target and receptor handle, degrader–target complexes are internalized from the plasma membrane into the endosomal system. This entry step is shared by both productive and non‐productive pathways. (B) Once internalized, complexes enter a competitive sorting network in which recycling, retrograde trafficking, transcytosis, non‐degradative retention and progression to late endosomes or lysosomes can compete. Productive degradation depends on commitment to degradative rather than recycling‐dominant routes. (C) Soluble extracellular cargo and membrane‐associated cargo impose different post‐endocytic requirements. Soluble cargo may be degraded after luminal delivery, whereas membrane proteins often require incorporation into multivesicular body or intraluminal vesicle pathways to enable destruction of transmembrane components. (D) Post‐endocytic fate can be tuned by receptor identity, target and receptor affinity, complex lifetime, valency, linker length and rigidity, pH responsiveness, molecular size and conjugation geometry. These features influence whether endosomal access becomes lysosomal commitment. (E) Failure modes include premature dissociation, rapid recycling, return of target to the cell surface, signalling without depletion and uptake without measurable total target loss. Accordingly, internalization should be treated as an intermediate step rather than as evidence of productive degradation.

In this Review, we consider lysosomal TPD as a problem of programmable endolysosomal routing (Figure 1D). Productive degraders must combine target engagement, selective cellular uptake and post‐endocytic sorting toward late endosomes and lysosomes; for transmembrane targets, they must also favor access to degradative intraluminal vesicle pathways (Figure 2A). We therefore examine the receptor and cargo features that govern this route, criteria for establishing lysosome‐dependent target degradation, and design principles for next‐generation degraders. In particular, we focus on actionable molecular cues—including receptor selection, degrader valency and geometry, receptor clustering, pH‐responsive interactions and sorting‐promoting trafficking mechanisms—that can reduce recycling and favor lysosomal delivery [27]. We further discuss how architecture, tissue exposure, pharmacokinetics and target biology should be matched to achieve selective and translatable degradation (Figure 1E). Although this Review focuses primarily on receptor‐recruiting chimeric degraders, lysosomal target loss does not always require an exogenous internalizing handle. Antibody‐mediated crosslinking, ligand‐induced receptor clustering and the intrinsic turnover of the target can promote internalization and subsequent lysosomal delivery. Antibody‐drug conjugate‐like architectures and direct target‐internalizing degraders therefore provide useful complementary examples. Their routing logic differs from that of classical LYTACs because productive uptake is determined by the target's own trafficking behavior or by target crosslinking rather than by recruitment of a separate clearance receptor. Including these systems broadens the framework from receptor hijacking to the more general problem of controlling target entry into degradative endolysosomal pathways.

## 2 Productive Lysosomal Degradation Requires Selective Capture, Uptake and Post‐Endocytic Sorting

Lysosomal degradation of extracellular and membrane‐associated proteins requires the coordinated optimization of three linked steps: selective target capture, receptor‐mediated uptake and post‐endocytic sorting toward lysosomes [28, 29]. Target binding and internalization establish entry into the pathway, but the degradative outcome is determined by whether target‐containing complexes avoid recycling, retrograde transport, and other non‐productive fates and instead progress through late endosomes to lysosomes. Thus, productive lysosomal TPD is not defined by uptake alone, nor by routing alone, but by successful integration of selectivity, cellular entry and degradative trafficking.

### 2.1 Selective Target Capture and Receptor‐Mediated Uptake Establish Entry

Target engagement must be sufficiently selective and compatible with the relevant extracellular, interstitial, or cell‐surface environment. For soluble proteins, this includes target abundance, accessibility and the availability of an internalizing clearance pathway. For membrane proteins, epitope position, target density, lateral mobility, baseline turnover and the geometry of receptor bridging can determine whether the target‐bound complex can be co‐internalized [30, 31, 32].

The recruited receptor or trafficking handle should be selected not simply for its expression level or uptake rate, but for its tissue distribution, endogenous ligand competition, safety profile, and post‐endocytic fate. Early LYTACs exploited the cation‐independent mannose‐6‐phosphate receptor (CI‐M6PR) [33], whereas subsequent liver‐directed platforms used the hepatocyte‐enriched asialoglycoprotein receptor (ASGPR) [34]. More broadly, receptors suitable for TPD should support target uptake without undesired receptor signaling or excessive competition with endogenous ligands [35]. Receptor density, internalization kinetics, recycling bias, and tissue‐specific expression together determine the attainable selectivity and efficiency of degradation [36].

### 2.2 Post‐Endocytic Sorting Determines Lysosomal Delivery

Following internalization, target–degrader–receptor complexes enter endosomes, where they can be recycled to the plasma membrane, transported retrogradely, transcytosed or delivered to late endosomes and lysosomes (Figure 2A). The balance between these fates is influenced by receptor identity, ligand geometry and valency, receptor clustering, endosomal acidification, binding kinetics, adaptor engagement, and sorting signals [37, 38]. These features provide actionable molecular cues for improving first‐generation degraders.

Several design principles follow. First, receptors with a favorable lysosomal‐delivery bias and limited recycling may be preferable to receptors chosen solely for rapid uptake [39]. Second, multivalent or geometrically optimized constructs can increase avidity and, where appropriate, promote receptor clustering and internalization; however, clustering should be used only when it enhances degradative sorting without activating the receptor or perturbing its physiological function [40]. Third, pH‐responsive binding can be tuned to enable receptor release and recycling in acidifying endosomes while retaining the target in the degradative itinerary. Finally, degrader architectures that favor recruitment of sorting machinery or exposure of lysosome‐directed signals may reduce return of target‐containing complexes to the cell surface.

Accordingly, trafficking measurements are most useful as design tools: they identify the steps at which a construct loses cargo to recycling or other competing routes and can guide changes in receptor ligand, epitope, linker, valency or binding kinetics. The functional endpoint, however, remains lysosome‐dependent loss of total target protein.

### 2.3 Additional Design Requirements for Membrane Proteins

Membrane proteins face an additional sorting requirement. After internalization, they remain in the limiting membrane of endosomes and generally must be incorporated into intraluminal vesicles of multivesicular bodies (MVBs) before efficient lysosomal degradation of the full‐length protein can occur (Figure 2B). This process can be influenced by receptor clustering, ubiquitin‐ and ESCRT‐associated sorting, membrane context, and adaptor recruitment [41, 42, 43].

For membrane targets, effective degrader design should therefore favor not only co‐internalization but also commitment to MVB sorting (Figure 2C). This consideration is particularly relevant to receptor tyrosine kinases, including EGFR, as well as immune checkpoints, adhesion receptors and transporters. In these settings, constructs that induce appropriate clustering or exploit transmembrane E3 ligase‐ and sorting‐associated processes may improve degradative routing, provided that they retain target and tissue selectivity and do not trigger unwanted receptor signaling [44, 45] (Figure 2D).

Together, these principles position lysosomal TPD as an engineering problem in which target selectivity, uptake route and intracellular sorting must be optimized as an integrated system. This framework informs both the mechanistic studies needed to refine degraders and the design strategies discussed in the following sections (Figure 2E).

## 3 An Evidence Framework for Lysosome‐Dependent Target Degradation

**FIGURE 3:**
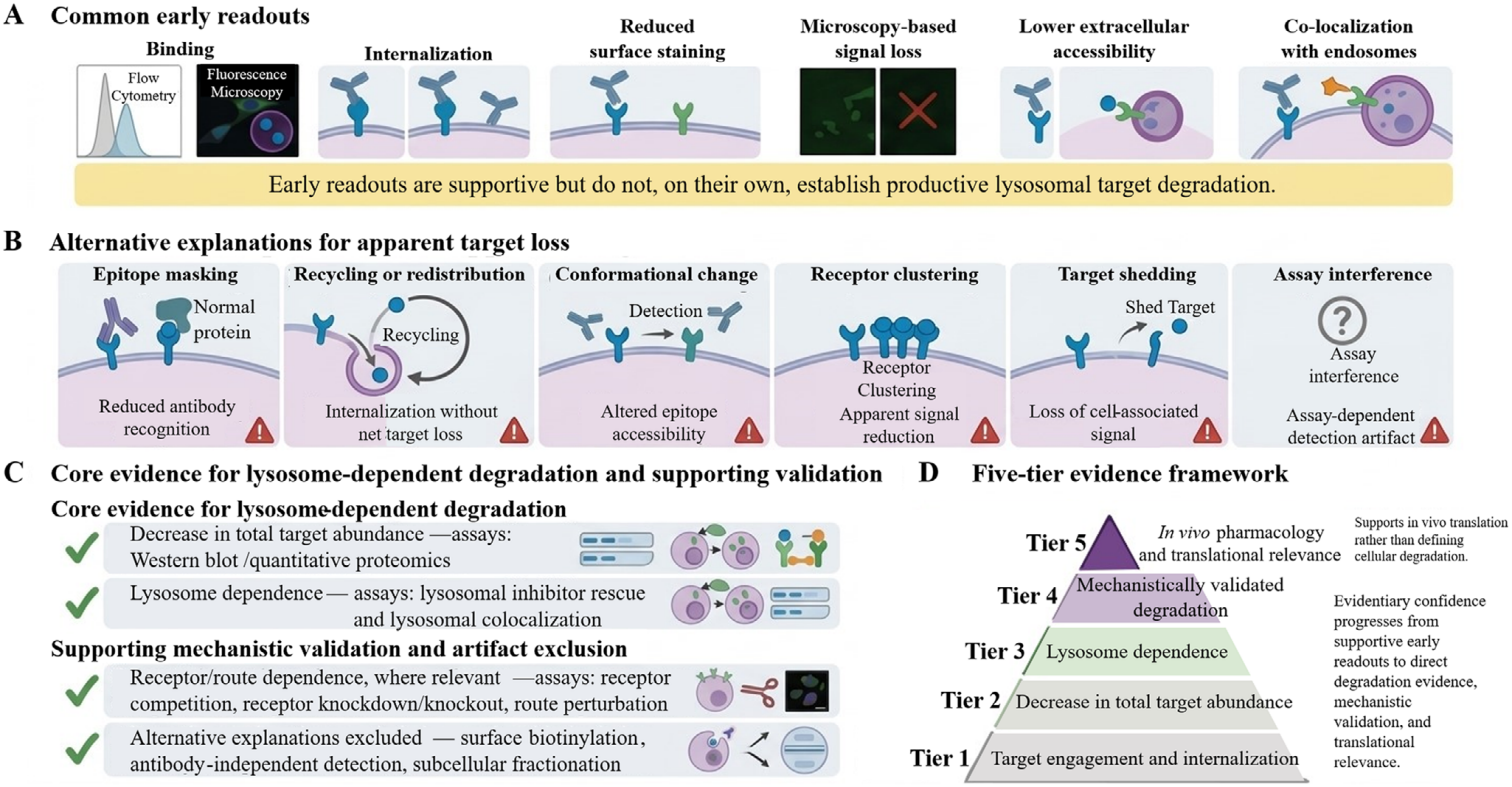
Distinguishing bona fide lysosomal degradation from permissive readouts and alternative explanations. (A) Common early readouts include target binding, co‐localization with endosomes, reduced extracellular accessibility, internalization, reduced surface staining and microscopy‐based signal loss. These measurements can support target engagement, cellular uptake or altered target accessibility, but they are permissive rather than definitive and do not, on their own, establish productive lysosomal target degradation. (B) Apparent target loss or signal reduction may arise from alternative mechanisms, including epitope masking, target shedding, assay interference, recycling or redistribution, receptor clustering, conformational change and altered antibody accessibility. These processes can reduce the measured signal without reducing total target abundance. (C) Core evidence for lysosome‐dependent degradation includes a quantitative decrease in total target abundance, measured by quantitative immunoblotting, proteomics or orthogonal methods, together with evidence of lysosomal dependence, such as rescue by lysosomal inhibition or perturbation. Supporting validation should establish dependence on the intended receptor or trafficking route and exclude alternative explanations using appropriate competition, genetic, trafficking, surface‐biotinylation, antibody‐independent detection and subcellular fractionation assays. (D) Five‐tier evidence framework comprising: Tier 1, target engagement and internalization; Tier 2, decreased total target abundance; Tier 3, lysosome dependence; Tier 4, mechanistically validated degradation; and Tier 5, in vivo pharmacology and translational relevance. The framework illustrates progression from supportive early readouts to direct degradation evidence, mechanistic validation, and translational assessment.

As targeted lysosomal degradation of extracellular and membrane‐associated proteins expands, clear criteria are needed to distinguish lysosome‐dependent target degradation from target binding, internalization, altered surface accessibility or changes in extracellular target detectability. Assays such as surface flow cytometry, fluorescence uptake measurements and microscopy are useful for characterizing target engagement and trafficking, but they do not alone establish target degradation (Figure 3A). The central experimental question is whether a degrader reduces total target protein through a lysosome‐dependent process in the relevant biological context.

### 3.1 Core Criteria for Lysosome‐dependent Target Degradation

Two criteria are central for concluding that a construct induces lysosome‐dependent target degradation. First, the construct should reproducibly reduce total target protein abundance. For membrane proteins, this requires measurement of total full‐length cellular protein rather than surface staining alone. For soluble targets, extracellular, cell‐associated and tissue‐associated pools should be distinguished where relevant. Orthogonal detection methods, including immunoblotting, quantitative capillary immunoassays, permeabilized flow cytometry, non‐competing immunoassays, targeted mass spectrometry or proteomics, can help establish genuine target loss and minimize errors arising from epitope masking, target redistribution or fragment detection [46, 47, 48, 49].

Second, target loss should show lysosomal dependence. Pharmacological perturbation of lysosomal acidification or proteolysis, using agents such as bafilomycin A1, concanamycin A, chloroquine, hydroxychloroquine or lysosomal protease inhibitors, can support this conclusion when it restores total target protein abundance. Because these interventions also affect endosomal trafficking, secretion, autophagic flux, signaling, and cell fitness, their interpretation should include viability controls and, where appropriate, target mRNA measurements and orthogonal protein assays [50, 51].

Dose‐response and time‐course studies further strengthen both criteria. Quantitative parameters such as DC50, Dmax and degradation kinetics define the magnitude and temporal profile of target loss, while washout and recovery experiments can distinguish durable protein depletion followed by resynthesis from transient redistribution or continued assay interference [52] (Figure 3B). Appropriate controls should also exclude broad cytotoxicity and major transcriptional or translational effects. These controls are necessary to interpret total target reduction correctly, but they should not obscure the primary conclusion: lysosome‐dependent loss of total target protein is the defining evidence for lysosomal TPD.

### 3.2 Mechanistic Analyses Strengthen Route Assignment and Degrader Optimization

Once lysosome‐dependent target loss has been established, additional experiments can define how a given degrader reaches this endpoint and identify opportunities for further optimization. For receptor‐recruiting systems, receptor dependence can be examined using receptor depletion, blocking antibodies, ligand competition, receptor‐negative vs. receptor‐positive cells, reconstituted systems or receptor‐binding‐defective degrader controls. These experiments support the proposed mechanism and establish whether the selected receptor is a productive trafficking handle, but they are not required to conclude that lysosome‐dependent target degradation has occurred.

Trafficking analyses, including pulse‐chase imaging, antibody‐feeding assays, surface biotinylation, endosomal fractionation and colocalization with early, recycling, late endosomal and lysosomal markers, can reveal whether target‐containing complexes are diverted toward recycling, retrograde transport or lysosomal delivery [53, 54] (Figure 3C). Such measurements are particularly useful for refining receptor selection, target epitope, linker properties, binding kinetics, valency and molecular geometry.

For membrane proteins, analysis of MVB sorting, ubiquitination, ESCRT dependence, receptor clustering and the fate of the recruited receptor may further explain differences in degradation efficiency [55, 56, 57]. These studies can identify whether a construct promotes degradative commitment or instead causes receptor co‐degradation, pathway perturbation or inefficient target recycling. Similarly, proteasomal or autophagy‐related controls may be informative where alternative routes are plausible. Collectively, these experiments strengthen mechanistic claims and guide the design of improved degraders; they should be presented as supporting evidence for route assignment rather than as universal requirements for establishing lysosome‐dependent target loss [58, 59].

### 3.3 In Vivo Validation Establishes Translational Relevance

In vivo studies should establish target reduction in the relevant tissue, cell type and anatomical compartment, together with an interpretable pharmacodynamic consequence. For soluble systemic targets, reduced plasma concentration may indicate target engagement or clearance, but direct evidence of target loss in the relevant biological compartment is more informative. For membrane targets in tumors or other heterogeneous tissues, measurements should, where feasible, distinguish target loss in the intended cells from changes in stromal, endothelial or immune compartments [60].

Receptor expression, target turnover, tissue penetration, degrader exposure, receptor occupancy and intracellular trafficking capacity are important variables for understanding in vivo performance and for improving degrader design. Likewise, tissue distribution, receptor perturbation, endogenous ligand competition, off‐tissue uptake, liver and spleen accumulation, immune activation and PK/PD relationships are essential considerations for translation and safety [61]. However, these parameters contextualize and optimize in vivo TPD; they do not replace demonstration of target reduction as the principal pharmacodynamic endpoint.

To facilitate comparison across target–construct–model combinations, we propose a tiered framework that distinguishes evidence for degradation from evidence that strengthens mechanistic interpretation or translational relevance (Figure 3D).

#### 3.3.1 Tier 1 Target Engagement and Uptake

The construct binds the target and/or enters cells. This establishes feasibility of target capture and delivery but does not demonstrate degradation.

#### 3.3.2 Tier 2 Total Target Protein Reduction

The construct reduces total target protein abundance in a reproducible, dose‐ and time‐dependent manner, supported by orthogonal detection and controls for assay interference, viability and major changes in target expression. This establishes biochemical target loss.

#### 3.3.3 Tier 3 Lysosome‐Dependent Target Degradation

Total target reduction is reversed or substantially attenuated by appropriate lysosomal perturbation, with controls supporting the interpretation of lysosomal involvement. This provides the key evidence for lysosome‐dependent TPD.

#### 3.3.4 Tier 4 Mechanistically Resolved Degradation

Receptor or route dependence, trafficking behavior, recycling, receptor fate and, where relevant, MVB sorting, ubiquitination or ESCRT involvement are characterized. This tier strengthens the mechanistic explanation and informs molecular optimization.

#### 3.3.5 Tier 5 Translationally Validated Degradation

Target reduction is demonstrated in relevant tissues or cell populations in vivo and is interpreted alongside exposure, PK/PD relationships, tissue selectivity, pharmacodynamic consequences and tolerability. This tier supports translational development and application.

The framework is intended to assess the evidence achieved for a specific target–construct–context combination rather than to assign an intrinsic maturity level to a platform class. Most importantly, Tier 2 and Tier 3 provide the essential evidence for biochemical target loss and lysosome‐dependent TPD, whereas Tier 1 establishes feasibility, Tier 4 strengthens mechanistic interpretation, and Tier 5 establishes translational relevance.

## 4 Receptor Handles Define the Opportunities and Limits of Programmable Lysosomal Routing

Receptor‐handle selection is a major determinant of extracellular and membrane‐targeted lysosomal degradation. However, handle abundance or internalization rate alone does not predict degradative performance. A useful handle must be evaluated according to its tissue distribution, endogenous function, ligand competition, post‐endocytic itinerary, capacity, safety liabilities and compatibility with the target and degrader architecture. In this Review, the term receptor handle encompasses cell‐surface receptors, clearance receptors, uptake routes and membrane‐associated degradation pathways that can be recruited by extracellular or membrane‐targeting degraders. These handles are not interchangeable entry portals: they provide distinct opportunities to control tissue selectivity, uptake and post‐endocytic sorting [62, 63, 64].

### 4.1 Lysosome‐Connected and Clearance Receptors

**FIGURE 4:**
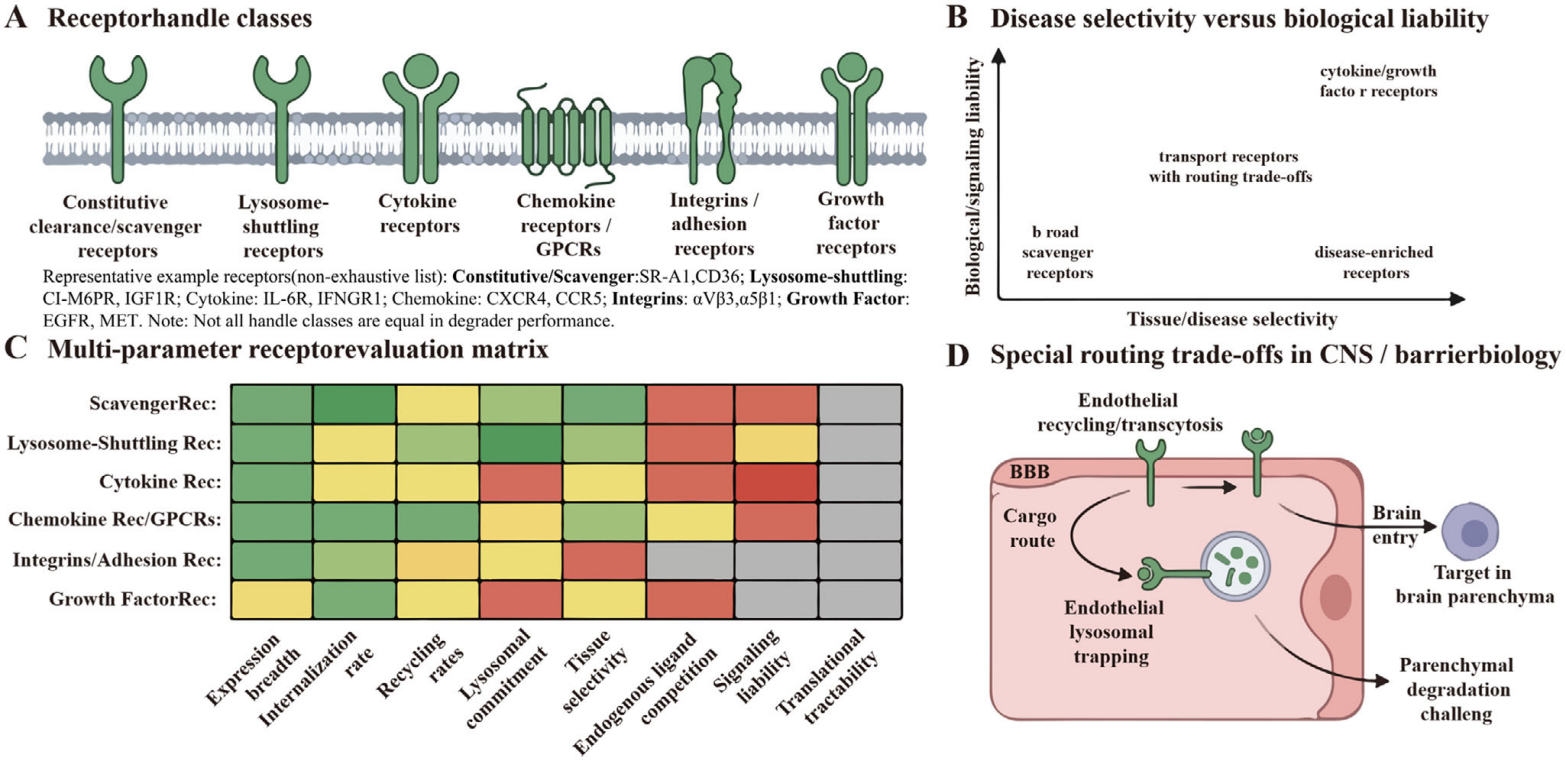
Receptor‐handle selection defines the opportunities and liabilities of programmable lysosomal routing. (A) Receptor handles used in targeted lysosomal degradation span multiple functional classes, including constitutive clearance or scavenger receptors, lysosome‐shuttling receptors, transport‐associated receptors, disease‐enriched internalizing receptors, and signalling‐active receptor families such as cytokine receptors, chemokine receptors, growth factor receptors, integrins, and immune receptors. These handles are mechanistically distinct rather than interchangeable cellular entry portals. (B) Receptor handles can be compared across parameters including expression breadth, internalization rate, recycling bias, lysosomal commitment, tissue selectivity, endogenous ligand competition, signalling liability, and translational tractability. Productive degradation cannot be predicted from uptake efficiency alone. (C) Receptor selection often involves a trade‐off between selectivity and biological liability. Broadly expressed clearance receptors may provide efficient uptake but limited tissue specificity, whereas disease‐enriched or signalling‐active receptors may improve selectivity at the cost of greater context dependence or pathway perturbation. (D) In specialised settings such as the central nervous system, receptor routes that favor endothelial uptake or barrier transit may not favor productive degradation in the intended parenchymal compartment. Receptor choice should therefore be matched to tissue context, trafficking logic and therapeutic objective.

The cation‐independent mannose‐6‐phosphate receptor (CI‐M6PR; also known as IGF2R) remains the prototypical handle for LYTAC design. Its physiological role in recognizing mannose‐6‐phosphate‐modified lysosomal enzymes and trafficking between the trans‐Golgi network, endosomes and lysosomes provided the conceptual basis for early LYTACs. By bridging extracellular or cell‐surface targets to CI‐M6PR through multivalent mannose‐6‐phosphate ligands, these constructs established that target engagement can be coupled to endogenous lysosomal‐trafficking machinery (Figure 4A).

CI‐M6PR is particularly useful when broad cellular accessibility and direct connection to lysosomal trafficking are desired. Its performance nevertheless depends on target topology, degrader geometry, ligand valency, receptor occupancy and post‐endocytic sorting. Endogenous ligand competition, receptor‐capacity constraints and possible perturbation of lysosomal enzyme trafficking should be considered during optimization, particularly for membrane targets that must ultimately enter degradative multivesicular‐body (MVB) pathways.

Sortilin and members of the low‐density lipoprotein receptor‐related protein family broaden the set of clearance‐ and sorting‐associated handles. Sortilin participates in trafficking among the plasma membrane, endosomes, lysosomes and the trans‐Golgi network [65], whereas LRP1 contributes to endocytosis, extracellular proteostasis, lipid metabolism and transport across cellular barriers, including at the blood‐brain barrier [66]. These receptors may be useful for extracellular cargo clearance, aggregate removal and disease settings in which their expression provides cellular or anatomical access. Their routing is ligand‐, adaptor‐ and cell‐context‐dependent, and should therefore be matched to the desired outcome, whether systemic clearance, cellular uptake, barrier transport or lysosomal degradation.

The asialoglycoprotein receptor (ASGPR) represents a distinct organ‐restricted clearance handle. Highly expressed on hepatocytes, ASGPR recognizes terminal galactose or N‐acetylgalactosamine residues and underlies the clinical success of GalNAc‐based liver delivery [67]. ASGPR‐based degraders are consequently attractive for plasma‐accessible soluble proteins, secreted mediators and extracellular targets that can be captured and cleared through hepatocyte uptake. In this setting, the liver can function as a selective clearance and degradative sink.

The principal advantage of ASGPR is liver selectivity, which can support repeated systemic target capture while limiting exposure in other tissues. Its utility depends on receptor recycling and capacity, ligand valency, endogenous glycoprotein competition, dosing regimen and liver health. For systemic soluble targets, plasma target reduction should be interpreted alongside tissue‐level pharmacodynamics and mechanistic evidence appropriate to the claimed mode of action.

### 4.2 Recycling and Transcytosis Handles Support Delivery and Require Routing Control for Degradation

TfR1 exemplifies a receptor that supports efficient uptake, recycling and, in selected settings, transcytosis. During iron acquisition, TfR1 undergoes clathrin‐mediated endocytosis and typically returns to the cell surface after endosomal iron release [68]. This behavior has made TfR1 a widely used delivery and blood‐brain‐barrier transport handle (Figure 4B). Its use for degradation requires a different design objective: target‐containing complexes must be biased away from recycling or transcellular transport and toward lysosomal commitment within the intended target cell.

Affinity, epitope, valency, avidity and pH‐dependent dissociation can influence this balance. Controlled receptor release in acidifying endosomes may preserve receptor recycling, whereas cargo retention, receptor clustering or an additional sorting‐promoting interaction may be required to favor target delivery to lysosomes. TfR1 should therefore be selected according to whether the primary need is cellular entry, barrier transport, target clearance or degradation in a defined cell type.

Integrins provide another useful class of uptake and localization handles. Their continuous internalization and recycling support adhesion, migration, and extracellular‐matrix remodeling [69], and their disease‐associated expression can provide access to tumors, invasive cells, and angiogenic vasculature. Integrin‐directed degraders may be valuable when tissue enrichment or cellular entry is the dominant requirement. Their design should account for receptor recycling and for possible effects on adhesion signaling, mechanotransduction, migration and survival.

More generally, recycling‐ and transcytosis‐prone handles can be converted into more productive degradation routes through optimized valency and geometry, controlled receptor clustering, pH‐responsive interactions, dual‐handle designs or coupling to sorting‐ and ubiquitination‐associated machinery [70, 71, 72, 73, 74, 75]. The appropriate strategy depends on the desired cellular itinerary rather than on uptake efficiency alone.

### 4.3 Disease‐Enriched, Growth Factor and Signaling‐Active Handles Provide Selectivity but Require Context‐Specific Control

Some handles are useful primarily because they provide disease, tissue or cell‐state enrichment rather than an intrinsic bias toward lysosomal routing (Figure 4C). Examples include prostate‐specific membrane antigen in prostate cancer, glypican‐3 in hepatocellular carcinoma [76], carbonic anhydrase IX in hypoxic tumors and renal cell carcinoma [77], folate receptor alpha in ovarian and other epithelial cancers [78], and selected integrins associated with tumor angiogenesis or invasion [79]. Such receptors can concentrate degraders in malignant tissue or enable tumor‐biased cellular entry. Their expression is often heterogeneous across patients, lesions, and tumor regions, so selective enrichment may need to be combined with an independent routing‐enhancing feature.

Growth factor receptors are an additional clinically relevant class for membrane‐targeted degradation. EGFR is a prominent example because it is dysregulated in multiple epithelial malignancies and has well‐characterized ligand‐ and antibody‐dependent endocytic trafficking. EGFR‐directed lysosomal degrader strategies may be valuable where sustained receptor removal provides advantages over transient occupancy‐based inhibition. Their design should account for receptor expression in normal epithelia, endogenous ligand competition, receptor recycling, endosomal signaling and the possibility that receptor clustering alters pathway activity or trafficking. More broadly, growth factor receptor biology can provide both disease‐relevant target classes and, in selected settings, useful trafficking logic for degrader development.

Cytokine receptors, chemokine receptors and G protein‐coupled receptors can likewise provide cell‐population selectivity in immune, inflammatory and endocrine settings. IL‐2R‐based targeting may distinguish lymphocyte subsets depending on receptor‐subunit usage and ligand design; CXCR7/ACKR3 has scavenging properties relevant to chemokine‐directed systems; and GLP‐1R provides an endocrine‐associated route supported by extensive clinical experience with GLP‐1‐based therapeutics [80, 81]. Because cytokines, chemokines, and peptide hormones are not inert recruitment ligands, their use can alter JAK‐STAT, PI3K‐AKT, MAPK, calcium, chemotactic, desensitization, or proliferative signaling [82].

Design strategies should therefore seek to preserve uptake while minimizing unwanted agonism and competition with endogenous ligands. Potential approaches include attenuated or antagonist‐like ligands, altered receptor‐subunit selectivity, affinity tuning, geometric control, and disease‐restricted activation. Signaling outputs, receptor fate, cytokine release, and cell‐subset effects should be monitored alongside total target loss.

Scavenger receptor‐associated uptake provides a broader clearance‐oriented option, particularly for aggregates, vesicles, nanomaterials and macrophage‐mediated removal [83]. These pathways can be useful where broad extracellular clearance is intended, but their promiscuity requires careful distinction between selective target routing and nonspecific mononuclear phagocyte‐system sequestration or inflammatory activation. Non‐targeted particle controls, target‐free comparators, biodistribution analysis, proteomic selectivity assessment and inflammatory profiling are particularly important for these platforms.

### 4.4 Controlled Clustering and Membrane E3‐associated Routes Enable Fate Engineering

Beyond receptor selection, molecular architectures can actively bias the fate of internalized target complexes (Figure 4D). Controlled multivalent engagement can increase local avidity and induce receptor clustering, thereby enhancing endocytic capture and, for suitable receptor systems, promoting retention in late endosomal and lysosomal pathways [84, 85]. Clustering should not be maximized indiscriminately: excessive receptor crosslinking can cause receptor downregulation, non‐physiological signaling, altered biodistribution, or nonspecific uptake. It is therefore best used as a tunable routing cue, optimized through ligand valency, spacing, geometry, and affinity [86, 87, 88, 89].

Membrane‐bound E3 ligases offer a mechanistically distinct strategy for fate engineering. By coupling target recruitment to ubiquitination‐dependent membrane sorting, they may promote ESCRT‐associated incorporation of transmembrane cargo into intraluminal vesicles of MVBs, a key step toward lysosomal degradation. RNF43 and ZNRF3 illustrate this principle because they regulate Wnt receptor abundance through ubiquitination‐dependent internalization and degradation [90, 91]. In appropriate cellular and molecular contexts, their recruitment may help redirect engineered target complexes toward degradative sorting rather than recycling [92].

Both clustering‐ and E3‐based strategies must be designed to minimize perturbation of endogenous receptor or signaling biology. For E3‐associated systems, relevant variables include ligase expression, target topology, degrader geometry and ubiquitin‐acceptor accessibility, as well as potential effects on ligase fate and Wnt‐associated signaling [90]. Target loss and lysosomal dependence should be assessed together with receptor or ligase fate, pathway perturbation and tissue tolerability. Ubiquitination, ESCRT involvement, MVB sorting and recycling assays can then identify whether a design has improved post‐endocytic commitment rather than simply increased uptake.

Across these handle classes, the key design question is not simply which receptor internalizes most efficiently, but which handle can achieve the desired combination of target selectivity, tissue access, uptake, lysosomal commitment and tolerability. Handle selection should therefore be matched to target topology, disease compartment, intended route of action and the molecular features available to control post‐endocytic fate.

## 5 Molecular formats shape target capture, trafficking and translational performance

**FIGURE 5:**
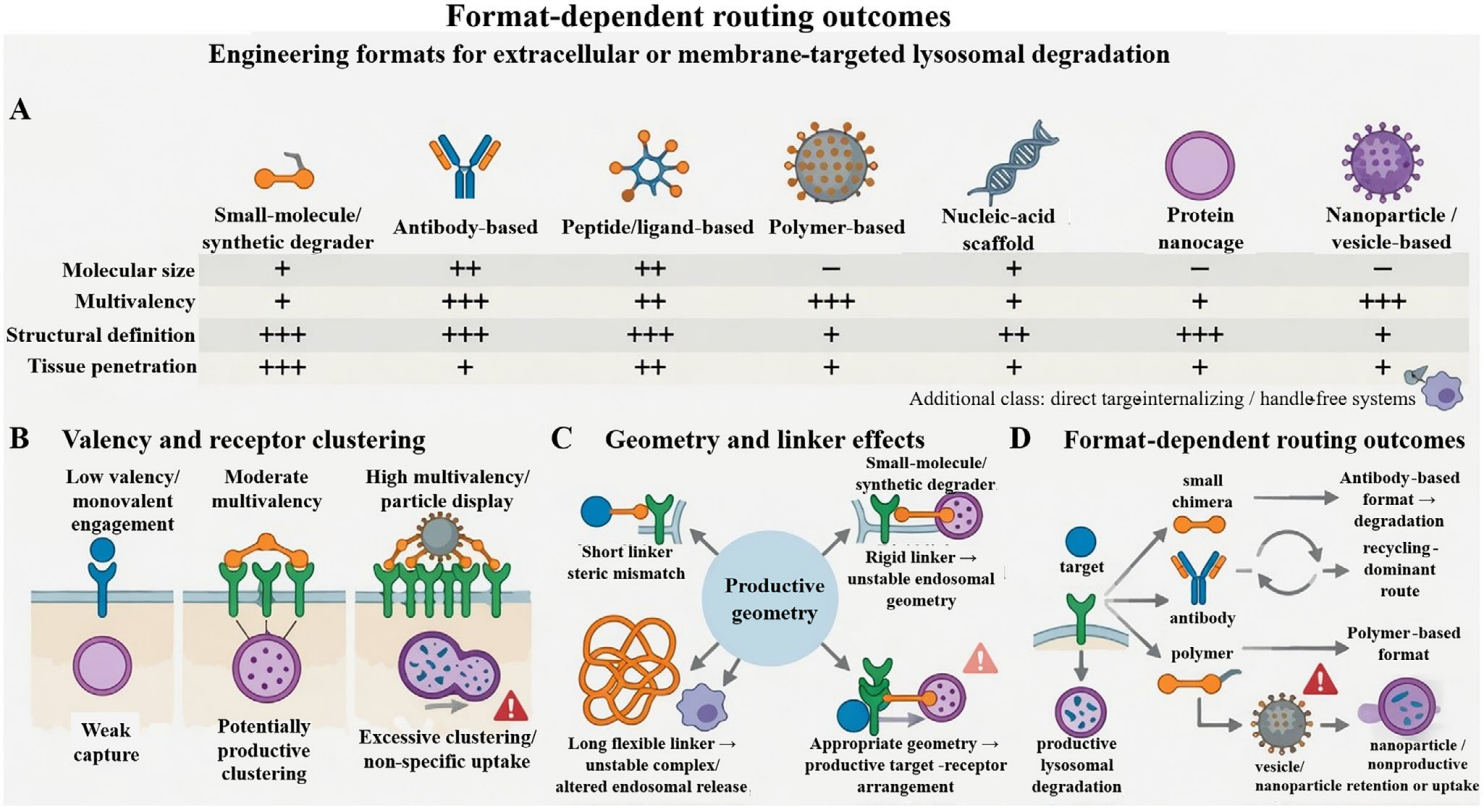
Molecular format, valency and geometry control trafficking fate and pharmacological behavior. (A) Targeted lysosomal degraders can be constructed in multiple formats, including small molecules or small proteins, nanoparticles or vesicle‐based systems, antibody‐based degraders, peptide‐ or ligand‐based systems and polymer‐based scaffolds. These formats span a broad range of molecular size, valency, structural definition and tissue‐penetration capacity. Additional classes include direct target‐internalizing or handle‐free systems that do not require a separate receptor‐recruiting component. Format‐dependent differences in size, architecture, flexibility, valency, and tissue distribution can influence target engagement, receptor recruitment, endocytic uptake, and intracellular trafficking. (B) Valency and receptor clustering can redirect post‐endocytic routing. Low‐valency or monovalent formats may produce limited receptor engagement and weak or non‐productive uptake, whereas moderate multivalency can promote productive target–receptor engagement and lysosomal delivery. Excessive valency or clustering, however, may result in non‐specific uptake, receptor sequestration, nanoparticle or degrader retention, unstable endosomal complexes, or other non‐productive outcomes. (C) Molecular geometry and linker properties further determine the productive engagement of target and receptor. Short or rigid linkers, long or flexible linkers, steric mismatch, inappropriate geometry, and altered endosomal release can affect complex stability, receptor clustering, target accessibility, and progression toward lysosomal degradation. Appropriate geometry and linker design are therefore required to balance target–receptor proximity, complex lifetime, intracellular release and productive lysosomal trafficking. (D) Format is therefore not a passive scaffold: identical target‐binding and receptor‐binding modules can produce different trafficking outcomes when displayed in different architectures. Mechanism, potency and translational behavior are jointly encoded by molecular format.

The molecular format of a lysosomal degrader determines how target recognition, receptor recruitment, valency, binding geometry, pharmacokinetics and tissue distribution are integrated into a functional degradation mechanism (Figure 5A). Early LYTACs were often conceived as bifunctional constructs in which one module binds an extracellular or membrane‐associated target and the other recruits an internalizing receptor. The field has since expanded to antibody conjugates, bispecific biologics, cytokine‐ and hormone‐based chimeras, compact synthetic degraders [93], multivalent assemblies, nucleic acid scaffolds, protein nanocages, vesicles and genetically encoded systems [94, 95, 96].

These formats differ not only in size and composition, but also in avidity, receptor clustering, endosomal dissociation, tissue penetration, serum persistence, manufacturability and immunogenicity. Format is therefore not simply a delivery scaffold. It can alter the probability that target engagement is converted into productive uptake and lysosomal delivery in a relevant tissue context. Architectural novelty alone, however, does not establish degradation; formats should be compared by their ability to produce total target loss with evidence of lysosomal dependence, together with an appropriate mechanistic and translational profile [94].

### 5.1 Modular Biologics Offer the Greatest Design Flexibility

Modular biologics remain the most established class of extracellular and membrane‐targeting lysosomal degraders. This group includes classical LYTACs, antibody‐ligand conjugates, bispecific antibodies, antibody fragments, nanobody‐based constructs and related bifunctional biologics that link a target‐binding module to a receptor‐recruiting ligand. Early CI‐M6PR‐ and ASGPR‐based LYTACs established the foundational principle that extracellular and cell‐surface targets can be connected to endogenous uptake and lysosomal‐trafficking pathways through induced proximity.

Their principal advantage is modularity. Antibodies and protein binders can provide high affinity and epitope selectivity, whereas the receptor‐recruiting component can be varied according to tissue access, receptor expression and trafficking behavior. Linker length, conjugation site, target epitope, receptor‐binding valency and overall stoichiometry can all influence ternary‐complex formation, receptor clustering, and post‐endocytic trafficking [95]. These variables make biologics particularly useful when the target lacks a tractable small‐molecule binder or when high selectivity is required.

Their limitations are equally important. Large constructs may show limited penetration into solid tumors or dense extracellular matrices and may have restricted access to protected anatomical compartments, including the central nervous system [96, 97]. Increased valency can enhance avidity and uptake, but excessive avidity or clustering may alter receptor fate, impair endosomal release, or promote undesired signaling. For IgG‐based constructs, Fc receptor engagement, immune‐complex behavior, and neonatal Fc receptor recycling can additionally influence biodistribution, persistence and uptake independently of the intended degrader mechanism [98]. Thus, the value of modular biologics lies in the opportunity to engineer these parameters systematically, rather than in any inherent guarantee of productive routing.

### 5.2 Signaling‐ligand Chimeras Can Provide Cell‐state Selectivity

Cytokine‐, chemokine‐ and hormone‐based chimeras use endogenous signaling ligands as targeting or uptake modules. By recruiting receptors such as IL‐2R, CXCR7/ACKR3 or GLP‐1R, these formats may direct activity toward immune subsets, inflammatory microenvironments, or endocrine tissues [99, 100]. Their major attraction is that they can exploit cell‐state or tissue selectivity that may be difficult to achieve using broadly expressed clearance receptors [82, 83, 84, 85, 86, 87, 88, 89, 90, 91, 92, 93, 94, 95, 96, 97, 98, 99, 100, 101, 102].

This selectivity is coupled to signaling liability. Cytokines, chemokines and peptide hormones can activate JAK‐STAT, PI3K‐AKT, MAPK, calcium, chemotactic, proliferative or immunomodulatory pathways, and may induce receptor desensitization, receptor downregulation or adaptive changes in cell state [103]. Such effects can be therapeutically useful in selected settings but may also confound mechanism or constrain dose.

Consequently, signaling‐ligand chimeras require design strategies that reduce unwanted agonism while preserving receptor engagement and uptake. Potential approaches include attenuated ligands, antagonist‐like binders, altered receptor‐subunit selectivity, affinity tuning, geometric control and pH‐responsive interactions. Evaluation should include signaling outputs, cytokine release, receptor fate and cell‐subset effects alongside measurements of total target loss [82, 83, 84, 85, 86, 87, 88, 89, 90, 91, 92, 93, 94, 95, 96, 97, 98, 99, 100, 101, 102, 103, 104].

### 5.3 Compact Synthetic Degraders Can Improve Tissue Access and Chemical Control

Small‐molecule, peptide and related compact synthetic degraders seek to reduce the size and structural complexity of larger biologics and multivalent assemblies. MoDE‐A‐like systems and related extracellular bifunctional molecules combine a compact target‐binding element with an uptake‐handle ligand, such as GalNAc, an M6P‐related ligand or a receptor‐binding peptide.

Compact formats may offer homogeneous chemical definition and greater control over linker length, rigidity, polarity, plasma stability and pH‐responsive binding. Their smaller size can also improve diffusion through tissues and access to constrained extracellular spaces, which may be advantageous where antibody penetration is limited [105, 106, 107, 108] (Figure 5B). These features can support iterative medicinal‐chemistry optimization and, depending on the molecular properties, alternative dosing or formulation strategies.

However, miniaturization does not eliminate the need for high‐quality target binding and productive receptor engagement. Many extracellular proteins lack suitable compact binders, particularly when accessible surfaces are large, flat or heavily glycosylated. In addition, small molecules and peptides generally lack the FcRn‐mediated recycling and long circulation time of IgG‐based constructs [109]. Rapid renal filtration, proteolysis and short in vivo half‐life can therefore limit sustained target capture and may require half‐life‐extension strategies, such as albumin binding, lipidation, PEGylation or long‐lived carrier conjugation. These modifications must be balanced against their potential effects on tissue penetration, receptor engagement and biodistribution. Compact degraders are thus chemically tractable architectures, but they must meet the same requirements for target selectivity, productive routing and in vivo exposure as larger biologics.

### 5.4 Multivalent and Advanced Platforms Expand Architectural Control

Polymeric scaffolds, glycopolymers, nucleic acid assemblies, nanocages, vesicles and genetically encoded systems can increase control over ligand number, spacing, stoichiometry and local concentration [110]. Their principal value is multivalent presentation, which can enhance avidity and promote receptor clustering when monovalent interactions are insufficient.

These advantages do not remove the need for target selectivity or productive lysosomal routing. Excessive multivalency or particle size can alter biodistribution, promote nonspecific uptake by scavenger or phagocytic pathways, and increase accumulation in clearance organs [111]. Genetically encoded systems additionally require control of delivery, expression level, reversibility and tissue restriction. Such platforms are therefore most useful when their additional architectural control produces a demonstrable gain in selective target loss and tolerability relative to simpler formats.

### 5.5 Molecular Cues Can Bias Internalized Complexes Toward Lysosomal Routing

Across formats, productive degradation depends on design features that bias target‐containing complexes away from recycling and toward late endosomes, multivesicular bodies (MVBs) and lysosomes (Figure 5C). Controlled multivalent clustering can enhance endocytic capture and, in suitable receptor contexts, favor degradative sorting. pH‐responsive interactions can permit selective dissociation in acidifying endosomes, allowing receptor recovery while retaining the target in a lysosome‐directed itinerary. Endosomal‐retention or sorting‐associated motifs may further reduce recycling and increase cargo residence in degradative compartments (Figure 5D).

For transmembrane targets, recruitment of membrane E3 ligases provides an additional route to fate control. Ligases such as RNF43 or ZNRF3 may, in appropriate cellular contexts, couple target recruitment to ubiquitination‐dependent ESCRT/MVB sorting and thereby increase lysosomal commitment. Because clustering, pH‐dependent release, sorting signals and E3‐ligase recruitment can also alter receptor fate, signaling and tissue distribution, they should be optimized together with target selectivity, exposure and tolerability. The key question is whether these features increase sustained total target loss with evidence of lysosomal dependence, rather than simply increasing uptake [110].

## 6 Design Principles for Productive Lysosomal Degradation

**FIGURE 6:**
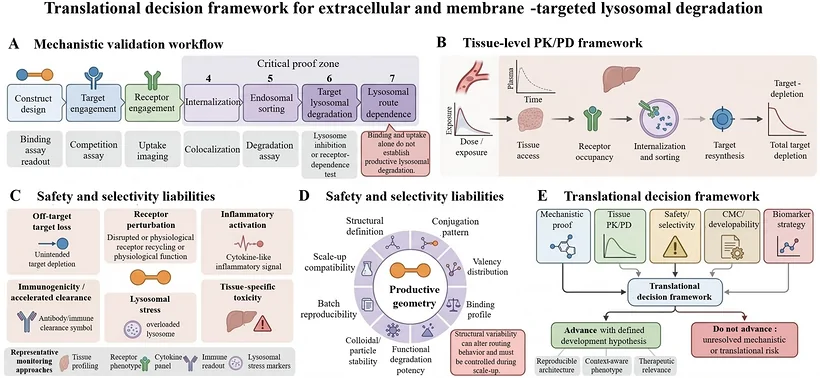
Translational decision‑making framework for extracellular and membrane‑targeted lysosomal‑degrading therapeutics. (A) Mechanistic validation workflow. step‑wise cascade from molecule construction, target and receptor engagement, cellular internalization, endosomal sorting, lysosomal degradation, to validation of lysosome‑dependent degradation; the critical proof zone highlights mandatory experiments to confirm productive lysosomal degradation, with corresponding functional readouts for each step. (B) Tissue‑level pharmacokinetic/pharmacodynamic (PK/PD) cascade. Schematic illustrating the sequential in‑vivo process: drug exposure, tissue penetration, receptor occupancy, endocytic internalization and sorting, target resynthesis, and final net target depletion. (C) Major safety and selectivity liabilities associated with lysosomal degraders, including off‑target degradation, receptor physiological perturbation, inflammatory activation, immunogenicity/accelerated clearance, lysosomal stress, and tissue‑specific toxicity, alongside representative monitoring biomarkers. (D) Key physicochemical and manufacturability factors governing productive molecular geometry, covering conjugation pattern, valency distribution, binding profile, functional degradation potency, colloidal stability, batch reproducibility, and scale‑up compatibility; structural heterogeneity may alter intracellular trafficking and must be tightly controlled during manufacturing. (E) Integrated translational decision framework. Five core evaluation pillars (mechanistic proof, tissue PK/PD, safety‑selectivity risk, CMC/developability, biomarker strategy) jointly inform a go/no‑go developmental decision: candidates meeting criteria proceed with a well‑defined working hypothesis, while molecules carrying unresolved mechanistic or translational risks are deprioritized.

The design of extracellular and membrane‐targeted lysosomal degraders should be guided by the complete target–construct–cell‐context combination rather than by target affinity or receptor internalization alone. The central optimization problem is to align target recognition, recruited‐handle behavior, post‐endocytic fate, tissue exposure and tolerability with the intended therapeutic objective (Figure 6).

### 6.1 Match the Recruited Handle and Exposure Profile to the Therapeutic Objective

Handle selection should follow the biological problem to be solved. High‐capacity clearance routes are well suited to plasma‐accessible soluble targets, whereas disease‐enriched or cell‐selective handles may be preferable for tissue‐resident membrane targets. The selected route must provide adequate tissue access, sufficient receptor capacity and an exposure profile compatible with target abundance, turnover and resynthesis (Figure 6B). In central nervous system applications, barrier transport and lysosomal routing in parenchymal target cells should be optimized as separate functions [112].

Format and pharmacokinetics must be considered at the same stage. Long‐lived biologics can support sustained target capture but may be limited by tissue penetration, whereas compact molecules may improve diffusion but require sufficient in vivo stability and half‐life. The required exposure profile depends on whether the therapeutic objective is repeated systemic clearance, sustained target depletion in a solid tissue or transient activity in a defined disease compartment.

### 6.2 Optimize Target Epitope, Molecular Geometry, Affinity and Valency

Target topology, extracellular‐domain accessibility, baseline trafficking and turnover should guide selection of the target‐binding epitope. For membrane targets, epitope location can influence steric compatibility, co‐clustering and endocytic capture, whereas binding to a single subunit may not necessarily remove an entire signaling complex [86, 87, 88, 89, 90, 91, 92, 93, 94, 95, 96, 97, 98, 99, 100, 101, 102, 103, 104, 105, 106, 107, 108, 109, 110, 111, 112, 113]. Linker length, rigidity, conjugation site and overall geometry should therefore be optimized empirically or structurally to support productive simultaneous engagement [87].

Neither affinity nor valency should be maximized indiscriminately. Binding must be sufficient for productive complex formation, but excessively stable interactions can saturate handles, impair receptor recovery, increase off‐tissue engagement or favor nonproductive trafficking. Similarly, multivalency can enhance avidity and capture but can also increase aggregation, nonspecific uptake and receptor perturbation. Matched affinity‐, valency‐ and geometry‐controlled comparisons are particularly useful for identifying designs that improve sustained target loss rather than uptake alone.

### 6.3 Bias Post‐endocytic Fate Toward Lysosomal Commitment

Post‐endocytic fate can be tuned through pH‐responsive dissociation, controlled receptor clustering, endosomal‐retention or sorting cues, dual‐handle architectures and, for suitable membrane targets, E3‐ligase‐associated sorting. pH‐dependent binding can allow recovery of the recruited handle while retaining cargo in a degradative itinerary. Controlled clustering, ligand spacing and geometry can enhance productive capture, but excessive crosslinking may alter receptor signaling or fate. For membrane targets that remain recycling‐prone after internalization, sorting‐promoting cues or recruitment of an appropriate membrane E3 ligase may further increase degradative commitment.

These features are optimization variables rather than universal mechanistic requirements. Endosomal binding and release, receptor recovery, washout response, recycling assays and total target‐loss kinetics can identify the post‐endocytic step that limits a given construct (Figure 6A).

### 6.4 Control Signaling and Restrict Activity Where Needed

When a recruited handle has endogenous signaling activity, degrader design should preserve uptake while minimizing unwanted agonism, pathway perturbation and competition with endogenous ligands (Figure 6C). Attenuated or antagonist‐like ligands, altered subunit selectivity, affinity tuning, geometric control and disease‐restricted activation can improve this balance. Evaluation should include relevant pathway activity, receptor fate and cell‐state effects alongside target reduction.

Disease‐site activation may further improve the therapeutic window where targets or handles are broadly expressed in healthy tissues. Protease‐, pH‐, hypoxia‐ or inflammation‐responsive masking strategies can restrict target binding, handle recruitment or multivalent assembly to diseased tissue [114, 115]. Their value depends on sufficiently selective activation and appropriate kinetics relative to local exposure.

Together, these principles provide a practical framework for optimizing first‐generation lysosomal degraders. The preferred architecture will depend on the dominant bottleneck in the intended setting—target accessibility, tissue exposure, uptake, recycling, post‐endocytic commitment, signaling liability or pharmacodynamic rebound—rather than on any single receptor or molecular format (Figure 6D,E).

## 7 Disease‐Matching Framework

The translational value of extracellular and membrane‐targeted degradation depends on whether target biology and disease context are compatible with the pharmacology of the chosen degrader. Rather than selecting a platform solely on the basis of target binding, development should begin by identifying the dominant requirement: sustained systemic clearance, selective removal of a tissue‐resident membrane protein, delivery across an anatomical barrier, avoidance of signaling perturbation, or rapid control of a dynamic extracellular target [116].

The suitability of a target for clearance‐based degradation is strongly influenced by its abundance, production rate, residence time and accessibility. Targets with very short circulating half‐lives may be less attractive for systemic depletion because endogenous renal or physiological clearance already limits their exposure. Such targets may nevertheless remain relevant when pathogenic activity depends on local production, protected tissue reservoirs or a membrane‐bound source. Conversely, highly abundant or rapidly replenished targets can impose a substantial stoichiometric and pharmacokinetic burden: sustained reduction may require high degrader exposure, sufficient recruited‐handle capacity and repeated uptake over time. Target concentration, turnover, receptor capacity, endogenous ligand competition and rebound after depletion should therefore be assessed early in development [117].

### 7.1 Requirements for Systemic Soluble‐target Clearance

Systemic clearance is most suitable for soluble targets that are accessible in plasma, lymph or interstitial fluid, persist long enough to be captured, and are not produced faster than the degrader system can remove them. High‐capacity uptake pathways and clearance organs are particularly valuable when repeated capture can drive cumulative target depletion. Development should quantify target abundance, production and resynthesis, degrader half‐life, handle occupancy, receptor recycling and clearance‐organ capacity. The relevant therapeutic question is whether the achievable exposure‐response relationship can overcome target replenishment without saturating the recruited pathway.

### 7.2 Requirements for Tissue‐resident Membrane Targets

Membrane‐targeted degradation is appropriate when target removal requires sustained depletion of a receptor, transporter, immune regulator or other surface protein within disease‐relevant cells. The principal requirements are selective target accessibility, adequate tissue penetration, uptake by the intended cell population and a molecular design that supports durable post‐endocytic commitment. Target shedding, heterogeneous expression, antigen sinks, receptor recycling and normal‐tissue expression can all reduce the therapeutic window. These settings therefore favor architectures that combine high target selectivity with sufficient local exposure and routing control.

### 7.3 Requirements for Targets Behind Anatomical Barriers

For targets in the central nervous system or other protected compartments, delivery to the relevant tissue and intracellular degradation within the target cell should be treated as separate design problems. A transport handle may enable barrier crossing but favor recycling or transcytosis rather than lysosomal delivery. Productive designs may consequently require staged, dual‐handle or otherwise compartment‐specific architectures that first achieve tissue access and then recruit a suitable degradation route in the intended parenchymal cell type [118, 119]. The required exposure should be evaluated in the target compartment rather than inferred from plasma pharmacokinetics alone.

### 7.4 Requirements for Signaling‐sensitive and Adaptive Biology

Targets and recruited handles in immune, inflammatory and endocrine settings require particular attention to biological compensation. Target depletion may alter feedback loops, receptor expression, cytokine production or endocrine homeostasis, while handle engagement can cause unwanted signaling or competition with endogenous ligands. These applications are best suited to constructs that provide strong cell‐type selectivity, controlled exposure, attenuated handle activity or disease‐restricted activation. Target reduction should be evaluated together with pathway activity, compensatory target resynthesis, cell‐state effects and long‐term tolerability.

### 7.5 Requirements for Rapidly Changing Extracellular Targets

Applications involving acute infection or rapidly evolving targets place greater emphasis on pharmacodynamic onset, target coverage and resistance resilience. A degrader must reach an effective concentration rapidly enough to compete with target production or pathogen replication, while retaining activity against target variation where relevant. Host‐directed approaches may reduce susceptibility to target escape but require a favorable safety margin because they perturb endogenous biology. These settings are most suitable when rapid exposure, flexible retargeting or broad target coverage can be achieved without unacceptable host‐pathway effects.

### 7.6 Matching the Dominant Bottleneck to the Degrader Strategy

Disease matching should ultimately be based on the step most likely to limit therapeutic activity. For soluble targets, the principal constraint may be target abundance, production rate or clearance capacity. For membrane targets, it may be tissue penetration, cell‐type access, receptor recycling or insufficient post‐endocytic commitment. For protected tissues, it may be compartmental delivery; for signaling‐sensitive biology, it may be pathway perturbation or pharmacodynamic rebound. This framework shifts platform selection from disease labels or format novelty toward a quantitative assessment of whether target exposure, degrader exposure, recruited‐handle capacity and biological tolerability can support sustained target loss.

## 8 Translational Challenges and Safety Considerations

Extracellular and membrane‐targeted degraders introduce translational requirements beyond those of conventional occupancy‐based inhibitors. Clinical development must account for perturbation of the recruited handle, tissue‐selective target loss, exposure‐dependent safety, immunological liability, manufacturability and biomarker‐based patient selection [120]. These considerations should be incorporated during lead selection because they can determine whether a construct with convincing cellular activity has a viable therapeutic window.

### 8.1 Recruited‐Handle Perturbation and Off‐Tissue Activity

Recruited receptors are physiological systems rather than passive uptake handles. Degrader exposure can compete with endogenous ligands, saturate receptor capacity, alter receptor recycling or signaling, and reduce handle availability through co‐internalization or co‐degradation. The magnitude of these effects may depend on dose, target burden, dosing frequency, disease‐associated changes in receptor expression, and the molecular geometry of the degrader.

Off‐tissue activity can arise when either the target or recruited handle is expressed in healthy tissues. Complex or multivalent formats may also undergo nonspecific uptake through scavenger pathways or mononuclear phagocyte‐system clearance. Safety assessment should therefore establish not only whether the intended target is degraded, but also where degradation occurs and whether recruited‐handle physiology is preserved. Receptor occupancy and recovery, tissue‐level target loss, proteomic selectivity, spatial pharmacodynamics and relevant pathway outputs should be assessed alongside conventional toxicology.

### 8.2 Exposure, Biodistribution and Tissue‐level Pharmacology

In vivo activity depends on whether degrader exposure, target abundance, recruited‐handle capacity and target resynthesis can support sustained target loss in the relevant tissue. Systemic pharmacokinetics alone may be insufficient to predict activity because local delivery, tissue penetration, receptor‐mediated clearance and spatial distribution can strongly influence both efficacy and toxicity. In dense tissues or protected compartments, limited activity may reflect inadequate delivery rather than a failure of target engagement or intracellular processing.

PK/PD analysis should therefore connect circulating and tissue exposure with target engagement, target reduction, recovery kinetics and distribution across intended and non‐intended tissues. These relationships are particularly important for targets with high abundance or rapid resynthesis, for which transient exposure or limited receptor capacity may prevent durable pharmacology.

### 8.3 Immunogenicity and Inflammatory Liability

Biologics, polymers, nucleic acid scaffolds, nanoparticles and vesicle‐based systems can introduce anti‐drug antibody responses, complement activation, cytokine release, innate immune sensing or inflammatory toxicity. Risk is influenced by molecular composition, multivalency, route and frequency of administration, patient population and disease state. It can be especially consequential for immune‐cell‐directed platforms or repeated dosing regimens.

Preclinical evaluation should include cytokine‐release, complement‐activation, innate‐immune activation and repeated‐dose tolerability studies, supplemented by anti‐drug antibody assessment where appropriate. Immunological liability can alter not only safety but also exposure, biodistribution and apparent pharmacodynamic activity.

### 8.4 Manufacturability and Quality Control

For complex degrader architectures, chemistry, manufacturing and control are closely linked to mechanism. Variation in conjugation site, ligand‐to‐carrier ratio, polymer length, valency, particle size, surface chemistry or vesicle composition can alter target binding, recruited‐handle engagement, tissue distribution, and degradation activity. A platform whose activity depends on a narrow structural window may be difficult to translate unless that window can be reproduced reliably at scale.

Quality attributes should therefore be defined using both structural and functional measurements. Depending on the platform, these may include composition, size or valency distribution, stability, receptor‐binding behavior, functional target‐degradation potency and properties predictive of in vivo exposure or biodistribution [121, 122].

### 8.5 Biomarkers and Patient Selection

Target expression alone is unlikely to be sufficient for patient selection. Response may depend on target accessibility, the abundance and functional capacity of the recruited handle, tissue exposure, target turnover and the distribution of both target and handle in normal tissues. Biomarker strategies should therefore integrate target biology with pharmacologically relevant trafficking and tissue context [123, 124, 125].

Spatial proteomics, surface proteomics, multiplex imaging, single‐cell profiling and functional pharmacodynamic assays can help identify tissues and patient subgroups in which the complete degradation pathway is accessible and tolerable. Such approaches may also identify predictable resistance mechanisms, including insufficient recruited‐handle capacity, unfavorable target turnover, or rapid pharmacodynamic rebound.

Together, these considerations define the translational threshold for extracellular degraders: a successful candidate must achieve selective and sustained target loss at exposures that preserve recruited‐handle function, maintain an acceptable safety margin, and can be reproducibly manufactured.

## 9 Conclusions and Outlook: From Receptor Hijacking to Programmable Endolysosomal Routing

### 9.1 Conclusions

Targeted lysosomal degradation extends targeted protein degradation beyond intracellular proteasome‐accessible substrates to secreted, extracellular, and cell‐surface proteins [126]. This expansion is therapeutically important because elimination of pathogenic mediators, extracellular aggregates and membrane receptors may provide pharmacology that cannot be achieved by transient occupancy‐based inhibition alone. LYTACs and related strategies have therefore established an emerging therapeutic interface between target recognition, cell‐surface trafficking and lysosomal biology.

A central conclusion from the first generation of these platforms is that internalization alone is not a sufficient endpoint. Productive activity depends on the fate of the target‐containing complex after uptake. The complex should avoid nonproductive recycling or redistribution and achieve sufficient lysosomal delivery to generate durable target loss. For membrane targets, surface removal does not necessarily predict degradation, because the subsequent post‐endocytic itinerary remains decisive [127]. Accordingly, degradation claims should be supported by evidence for total target loss, lysosomal dependence and, where relevant, the route by which the target is committed to degradation.

This Review proposes a routing‐centred framework for comparing and designing extracellular and membrane‐targeted degraders. Recruited handles should be viewed not as interchangeable entry portals, but as physiological systems that determine tissue access, uptake capacity, post‐endocytic fate and potential safety liabilities [128]. Platform performance should similarly be assessed in the context of the target–construct–cell combination, including target topology, molecular geometry, affinity and valency, tissue exposure, target turnover and post‐endocytic routing behavior.

Clinical translation will require the same mechanism‐guided perspective. Receptor perturbation, endogenous‐ligand competition, off‐tissue target loss, immunogenicity, manufacturability and tissue‐level PK/PD should be considered alongside cellular degradation potency. Biomarkers should integrate target expression with recruited‐handle capacity, tissue accessibility, target turnover, and pharmacodynamic evidence of target loss in the relevant compartment.

Together, these principles define the transition of the field from proof‐of‐concept target internalization to controllable extracellular and membrane protein elimination. If rigorous validation, routing‐aware molecular design and disease‐matched development are integrated, targeted lysosomal degradation may become a broadly useful strategy for programmable control of extracellular and cell‐surface proteostasis.

### 9.2 Future Perspectives

Over the next decade, progress will likely depend on moving beyond receptor hijacking toward more precise control of endolysosomal routing. Molecular architectures may increasingly encode post‐internalization behavior through affinity tuning, pH‐responsive dissociation, switchable or asymmetric valency, controlled clustering, disease‐site activation, and dual‐handle strategies [129]. These approaches may help balance target capture, receptor recycling, endosomal retention, and lysosomal commitment, while preserving the physiological function of the recruited handle.

Receptor engineering may provide an additional route to improve selectivity and routing control. Synthetic trafficking receptors or engineered receptor variants could, in principle, separate target capture from tissue delivery and lysosomal commitment [130]. Similarly, membrane‐associated E3 ligases may offer complementary mechanisms for coupling extracellular target engagement to ubiquitination‐dependent internalization or sorting [131]. The utility of such approaches will depend on ligase expression, target topology, cell type, and the extent to which the induced trafficking route produces durable target loss.

Computational and data‐driven design should also accelerate platform optimization. AI‐assisted structural modelling and multiparametric screening may help identify productive target epitopes, receptor combinations, linker architectures, valency, and geometry. Importantly, these approaches should aim to optimize not only binding affinity or internalization, but also the trafficking outcomes that determine degradation. Predictive models will nevertheless require experimental validation because endosomal routing remains highly dependent on cell type, receptor physiology and disease state.

Spatial and single‐cell technologies may provide the translational measurements needed to support this next phase. Spatial proteomics, surface proteomics, multiplex imaging and single‐cell degradation profiling can determine whether the target, recruited handle and degradative machinery coexist in the same disease‐relevant cells and anatomical compartments. Such analyses may distinguish delivery failure from routing failure, identify patient subgroups with sufficient handle capacity and enable more informative pharmacodynamic monitoring in vivo.

Finally, receptor‐recruiting and handle‐free strategies should be assessed within a shared mechanistic framework. In some settings, target‐intrinsic turnover, ligand‐induced internalization or antibody‐mediated crosslinking may provide a more suitable route to lysosomal loss than recruitment of an external clearance receptor. Comparing these approaches according to target accessibility, internalization mechanism, post‐endocytic fate, total target loss and safety may clarify which strategy is best matched to each therapeutic problem.

Ultimately, the field must move beyond asking whether a target can be internalized and instead learn to control its intracellular fate. This shift—from uptake to routing, from receptor recruitment to itinerary programming, and from platform invention to mechanism‐guided design—will define the next stage of targeted lysosomal degradation.

## Author Contributions

K.L., X.W., X.M., and L.Z. wrote the manuscript and created the figures. X.L., C.H., and D.Q. created the figures. K.L., X.W., D.Q., X.M., and L.Z. conceived the final approval of the version to be submitted. All authors read and approved the final manuscript.

## Funding

This research was supported by the National Natural Science Foundation of China (Grant No. 82404685), Zhejiang Science and Technology Department “vanguard” “leading goose” research (Grant No. 2023C03044), and Zhejiang Province Traditional Chinese Medicine Science and Technology Plan Project (No. 2026ZL0193).

## Conflicts of Interest

The authors declare no conflicts of interest.

## Data Availability

Data sharing not applicable to this article as no datasets were generated or analysed during the current study.
